# AtomicChargeCalculator: interactive web-based calculation of atomic charges in large biomolecular complexes and drug-like molecules

**DOI:** 10.1186/s13321-015-0099-x

**Published:** 2015-10-22

**Authors:** Crina-Maria Ionescu, David Sehnal, Francesco L. Falginella, Purbaj Pant, Lukáš Pravda, Tomáš Bouchal, Radka Svobodová Vařeková, Stanislav Geidl, Jaroslav Koča

**Affiliations:** CEITEC-Central European Institute of Technology, Masaryk University, Kamenice 5, 625 00 Brno, Czech Republic; National Centre for Biomolecular Research, Faculty of Science, Masaryk University, Kotlářská 2, 611 37 Brno, Czech Republic; Faculty of Informatics, Masaryk University, Botanická 68a, 602 00 Brno, Czech Republic

**Keywords:** Conformationally dependent atomic charges, Biomacromolecules, Drug-like molecules, Paracetamol, Benzoic acids, Protegrin, Proteasome, Allostery, Chemical reactivity

## Abstract

**Background:**

Partial atomic charges are a well-established concept, useful in understanding and modeling the chemical behavior of molecules, from simple compounds, to large biomolecular complexes with many reactive sites.

**Results:**

This paper introduces AtomicChargeCalculator (ACC), a web-based application for the calculation and analysis of atomic charges which respond to changes in molecular conformation and chemical environment. ACC relies on an empirical method to rapidly compute atomic charges with accuracy comparable to quantum mechanical approaches. Due to its efficient implementation, ACC can handle any type of molecular system, regardless of size and chemical complexity, from drug-like molecules to biomacromolecular complexes with hundreds of thousands of atoms. ACC writes out atomic charges into common molecular structure files, and offers interactive facilities for statistical analysis and comparison of the results, in both tabular and graphical form.

**Conclusions:**

Due to high customizability and speed, easy streamlining and the unified platform for calculation and analysis, ACC caters to all fields of life sciences, from drug design to nanocarriers. ACC is freely available via the Internet at http://ncbr.muni.cz/ACC.

**Electronic supplementary material:**

The online version of this article (doi:10.1186/s13321-015-0099-x) contains supplementary material, which is available to authorized users.

## Background

Partial atomic charges are real numbers meant to quantify the uneven distribution of electron density in the molecule, and have been used for decades in theoretical and applied chemistry in order to understand the chemical behavior of molecules. Atomic charges are extensively used in many molecular modeling and chemoinformatics applications. With respect to biomacromolecules, charges can elucidate electrostatic effects critical for long range molecular recognition phenomena, protein folding, dynamics and allostery, directed adduction of substrates and egression of products in enzymes, ligand binding and complex formation for proteins and nucleic acids, etc. [1–3]. With respect to drug-like molecules, atomic charges provide information related to reactivity and can be used in the prediction of various pharmacological, toxicological or environmental properties [4, 5].

Although, in principle, it is possible to estimate atomic charges based on experimental measurements (e.g., [6, 7]), such calculations are impractical. Most commonly, atomic charges are estimated based on theoretical approaches. Quantum mechanical (QM) approaches first solve the Schrödinger equation [8] and calculate the electron density using a combination of theory level and basis set. They then partition the obtained molecular electron density (or a density-derived quantity) into atomic contributions (atomic partial charges) according to various population analyses [9–19]. Empirical approaches to atomic charge calculation (e.g., [20–27]) have been proposed as resource-efficient alternatives to QM approaches, as they do not require the demanding step of solving the Schrödinger equation. In particular, approaches based on the equalization of molecular electronegativity [22, 23, 28–35] are of interest because they are sensitive to both the chemical environment and molecular conformation.

Due to the essential role of atomic charges, many modeling tools currently include atomic charge calculation capabilities (e.g., [36–50]). However, in the case of drug-like molecules, only a few tools can provide QM quality charges which respond to changes in conformation or chemical environment without needing to first obtain the QM electron density or electrostatic potential [47, 48, 50]. Moreover, these tools are not sufficiently general, resource-efficient or interactive. In the case of biomacromolecules, no freely available software tool can readily provide atomic charges of QM quality, despite repeated reports that such quality is necessary [51–54]. We have accepted these challenges and set out to provide a robust and accessible software solution for atomic charge calculation for molecules of all nature and size.

This contribution presents the AtomicChargeCalculator (ACC), a free web application for the calculation and analysis of atomic charges which respond to changes in molecular conformation and chemical environment. The calculation is based on the electronegativity equalization method (EEM [22]), a powerful empirical approach which can provide atomic charges similar to those generated by various QM approaches, but using much lower computational resources. Along with the classical EEM algorithm, ACC implements two additional EEM approximations with increased efficiency, specifically tailored for studying very large molecular systems. A single calculation may take from less than a second (small molecules), to a few minutes (large biomacromolecular complexes). ACC outputs the most common molecular structure formats containing atomic charges. Additionally, it provides facilities for statistical analysis and comparison of the results, in tabular and graphical form. ACC also includes interactive 3D visualization of the molecules based on atomic charges. A command line version is also available.

## Implementation

The challenge was to provide a robust web based software solution for atomic charge calculation for molecules of all nature and size. Therefore, we first focused on identifying and optimizing a suitable algorithm for atomic charge calculation, and then on implementing the optimal workflow for setting up an ACC calculation and interpreting the results.

**Fig. 1:**
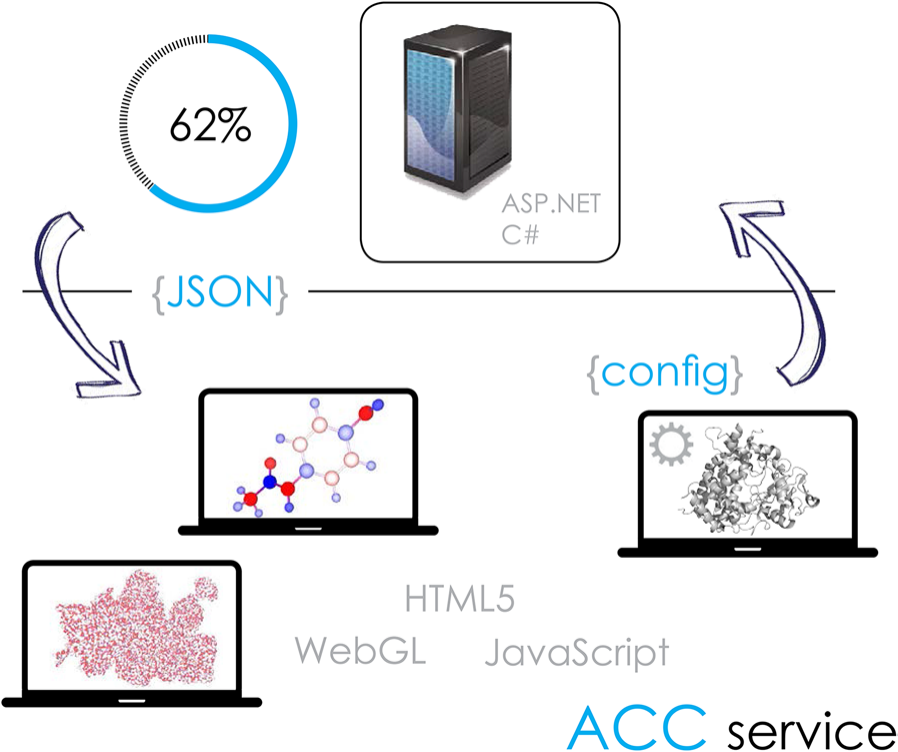
ACC application architecture. The client allows the user to setup the calculation via the user interface. The settings are sent to the web server in the form of a configuration file. The charge computation takes place on the web server. The results are sent back to the client for visualization and download by the user

The application was constructed using the client-server architecture (Fig. 1): the charge computation is carried out on the server and implemented in the C# programming language. The JavaScript Object Notation (JSON) is used to transfer data to the client that provides the user interface (UI) implemented using HTML5 and JavaScript. Additionally, the UI uses the WebGL technology to provide a custom built 3D visualization of the computed charges.

### Computational details

The Electronegativity Equalization Method (EEM) is the general approach followed by ACC to calculate atomic charges. EEM-based methods have been successfully applied to zeolites and metal-organic frameworks, small organic molecules, polypeptides and proteins [55–61].

EEM is an empirical approach which relies on parameters usually fitted to data from reference QM calculations. The values of atomic charges computed using EEM support chemical reasoning, and generally correlate well with values from reference QM calculations. The accuracy of each set of EEM parameters is documented in the respective literature. On the other hand, classical EEM approaches incorrectly predict superlinear scaling of the polarizability with increasing molecular size, making the models developed on small molecules difficult to transfer to extended systems like biomacromolecules [62, 63]. This artifact can be tempered by applying charge conservation constraints to small molecular units. Such extensions to EEM have been proposed [24, 64].

Computationally efficient implementations of EEM-based methods are integrated in tools specialized for reactive molecular dynamics simulations [65] and for generating conformers of drug-like molecules [50, 66]. However, ACC is the first to implement EEM in a manner which is not only computationally efficient, but also independent of the subsequent intended application, and specifically designed to allow users with little background in computational sciences to run charge calculations and interactively analyze the results.

ACC can solve the EEM matrix equation (see the Computational details section of the Additional file 1) if the following input is provided: the 3D structure of the molecular system, the total molecular charge, and a set of EEM parameters. Solving the EEM matrix equation requires solving a dense system of equations. The computational complexity of this procedure is $\mathcal {O}(N^3)$. The space complexity, which refers to the memory required to store the EEM matrix, is $\mathcal {O}(N^2)$, where *N* is the number of atoms. For very large molecules with tens of thousands of atoms, the EEM approach is too demanding on conventional desktop hardware. We thus propose two new approaches for solving the EEM matrix, namely EEM Cutoff and EEM Cover. These approaches work by splitting the EEM matrix into multiple smaller matrices.

Within the EEM Cutoff approach, for each atom in the molecule, ACC generates a fragment made up of all atoms within a cutoff radius *R* of the original atom. Thus, for a molecule containing *N* atoms, the EEM Cutoff approach solves *N* smaller EEM matrices, for a set of *N* overlapping fragments of the original molecule. EEM Cutoff effectively reduces the time complexity of the calculation to $\mathcal {O}(R^6 N + R^2 N log N)$, and the space complexity to $\mathcal {O}(R^4 N + N log N)$. A detailed description of the EEM Cutoff approach is given in the Computational details section of the Additional file 1.

To further enhance the run-time and memory efficiency of calculations in ACC, we propose EEM Cover, an approach for tackling molecules with hundreds of thousands of atoms. EEM Cover also splits the EEM matrix into smaller matrices, but it generates fragments only for a subset of atoms in the molecule. While the asymptotic complexity remains the same, the number of EEM matrices that need to be solved is reduced by at least 50 % compared to EEM Cutoff, while maintaining high accuracy. A detailed description of the EEM Cover approach is given in the Computational details section of the Additional file 1.

### Workflow

**Fig. 2:**
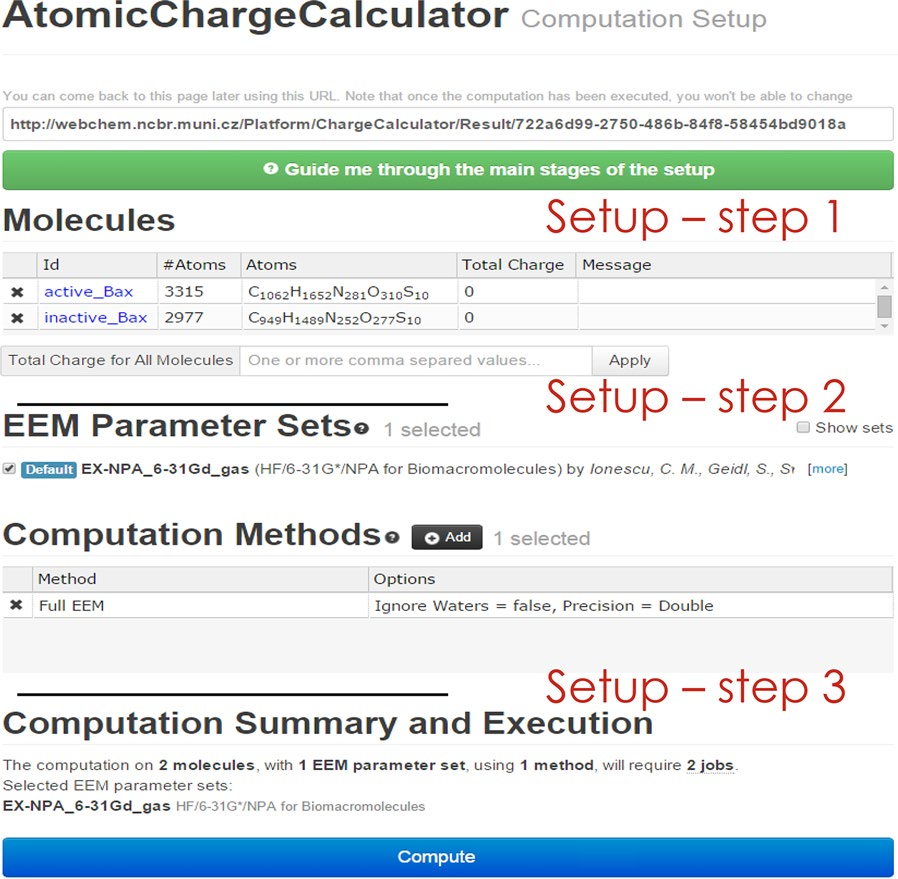
Setup of jobs in AtomicChargeCalculator. The setup of an ACC calculation takes place in three steps, each step referring to one of three aspects: the molecule and its total charge, the set of EEM parameters to be used in the EEM equation, and the computation options. These three aspects uniquely define an ACC job. A single setup may lead to running several ACC jobs. Based on the information in the uploaded structure files, ACC suggests a default setup, which can be adjusted by the user prior to starting the calculation. Explanations are available in the interactive guides, tool tips and Wiki pages

**Fig. 3:**
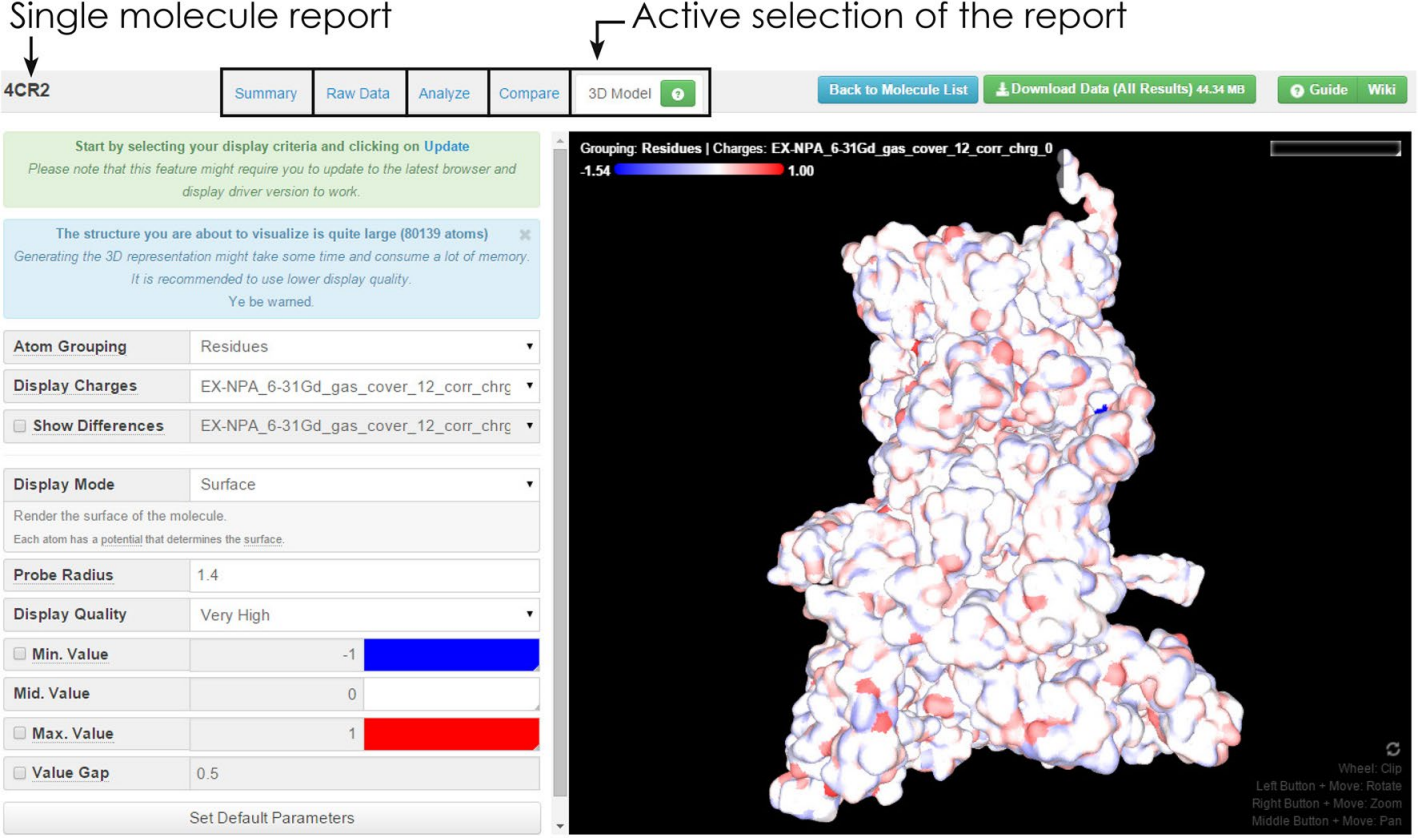
Single molecule report provided by AtomicChargeCalculator. It can be downloaded or inspected in the browser, and consists of several sections: summary information about the molecule, interactive list of values for all sets of charges, statistics within each set of charges, pairwise comparison statistics between sets of charges, and interactive 3D visualization of molecules

The ACC workflow is organized into four phases, namely: upload, setup, calculation and results. Each phase is characterized by a set of operations as follows:

1. *Upload molecules* Multiple molecules can be uploaded in the most common file formats (PDB, PDBx/mmCIF, PQR, MOL, MOL2, SDF, or .zip with multiple files of a suitable format). The molecular structures should be complete and properly protonated. There is no limitation regarding the size, number or nature of the chemical entities in a single structure file (proteins, nucleic acids, ligands, water, etc.), as all these are loaded and identified as a single molecule within ACC. The total size of the upload is limited to 50 MB.
2. *Setup* Upon uploading the molecule(s), ACC parses the molecular structure to identify the number and types of atoms in the system, as well as the inter-atomic distances. Based on this information, ACC tries to prefill the submission form with suitable default settings (see the Default settings section of the Additional file 1). These settings can be adjusted by the user before the calculation is started. Each distinct setup (Fig. 2) will result in a certain number of ACC jobs, each defined by the molecule, total molecular charge, the set of EEM parameters, and the computation options. For the command line version of ACC, the setup workflow is identical to the steps described below, and is scripted into a configuration file.
  2.1. *Total molecular charge* The total molecular charge quantifies the amount of charge that will be distributed across the molecule during the EEM calculation. By default, ACC assumes that all uploaded molecules are neutral. The user must provide the correct total molecular charge for each non-neutral molecule uploaded.
  2.2. *Set of EEM parameters* EEM employs special parameters for each type of atom (H, C, N, O, halogens, metals, etc., depending on the target molecules). EEM parameters are generally developed based on reference QM calculations. The applicability domain of a given EEM parameter set is generally limited to the target molecules, and closely related to the applicability domain of the particular QM approach used as reference. Performance is further influenced by the procedure used when fitting the EEM parameters to the reference data. Many EEM parameter sets have been published in literature, and are available in ACC as built-in sets [28, 34, 67–70] with full information regarding the parameter development procedure (atom types covered, target molecules, QM reference data, literature reference). By default, ACC tries to select one of these sets based on the atom types present in the uploaded molecules. The user can select a different set of EEM parameters by choosing from the list of available built-in sets, or even uploading customized sets in an XML template. Multiple sets of EEM parameters can be tested in a single ACC run.
  2.3 *Computation options* ACC may compute atomic charges based on one of the three available EEM approaches implemented, namely Full EEM, EEM Cutoff, and EEM Cover. Further options refer to the precision (64 or 32-bit representation of numbers), cutoff radius parameter, and including water molecules into the calculation. By default, ACC picks computation options most suitable to the size of the uploaded molecules. These computation options can be adjusted by the user. Up to 10 computation options can be tested in a single ACC run.
3. *Calculation* Once the setup phase is complete, the calculation is launched. A single ACC run may consist of multiple atomic charge calculation jobs. Each job is uniquely defined by the molecule, total molecular charge, set of EEM parameters, and computation options, and produces one set of atomic charges. Each job may use a different amount of time and memory resources, depending on the size of the molecule and the complexity of the computation.
4. *Results* The ACC results are organized into hierarchical reports which are stored on the server for download or inspection for up to a month, at a unique URL visible only to the user. The command line version of ACC produces the same overall and single molecule reports described below, but does not facilitate interactive 3D visualization.
  4.1. The *overall report* contains information and downloadable content (molecular structure files containing atomic charges, statistics of the results, information about all jobs) for all molecules. Single molecule reports are also accessible from here.
  4.2. The *single molecule report* (Fig. 3), which can be downloaded or examined directly in the browser, consists of a few sections:
    4.2.1. *Summary report* containing general information about the input molecule (molecular formula, total charge), calculation setup, a list of all sets of charges produced during the calculation, information about all ACC jobs (duration, warnings, errors) for that molecule.
    4.2.2. *Interactive list of values* for all sets of charges produced by all ACC jobs for that molecule. The atomic charges and residue charges are given.
    4.2.3. *Statistics within each set of charges*, in both tabular and graphical form. The statistics are available for both atomic and residue charges, and are computed for relevant properties such as chemical element, type of residue, etc. The statistical indicators are the minimum value, maximum value, standard deviation, average, median, etc.
    4.2.4. *Pairwise comparison statistics* between sets of charges resulted from different ACC jobs, or uploaded by the user. A graphical representation for each comparison is also provided. The comparison is available for atomic and residue charges. The comparison indicators computed are the squared Pearson’s correlation coefficient, Spearman’s rank correlation coefficient, RMSD, sum of absolute differences.
    4.2.5. *Interactive* 3D visualization of molecules. The 3D model can be built based on atomic positions, and colored based on atomic charges, or built based on residue positions, and colored based on residue charges. The coloring scheme can also use differences in charges resulted from distinct ACC jobs, or uploaded by the user.

The applicability of ACC is limited by three main aspects: related to the concept of atomic partial charges and its definitions, related to the concept of EEM and its parameters, and related to the 3D structure of the molecule and its total charge. These aspects are discussed in detail in the Limitations section of the Additional file 1.

Full documentation explaining the methodology, functionality and interface, along with interesting examples are provided on the web page. Embedded interactive guides assist first-timers and beginners in setting up their calculations and interpreting the results. A command line version of the application is available as an executable for users who wish to streamline more complex calculations.

## Results and discussion

### Implementation benchmark

**Fig. 4:**
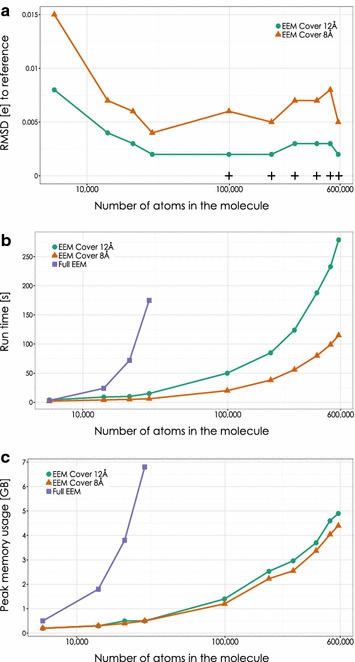
Benchmark of the EEM Cover approach. Full details can be found in the Benchmark section of the Additional file 1. **a** EEM Cover is sufficiently accurate, and its accuracy increases with the value of the cutoff radius. The root mean square deviation (RMSD) is calculated by comparison against the reference calculation which solves the entire EEM matrix (Full EEM), using 64-bit precision numbers. Due to the limitation on computational resources, for some molecules the reference calculation was *EEM Cutoff, with a cutoff radius of 17 Å. **b** EEM Cover is significantly faster than Full EEM only for molecules with over 10,000 atoms. **c** EEM Cover is always more memory efficient than Full EEM

We have evaluated the accuracy and computational efficiency of the EEM Cutoff and EEM Cover approaches in a benchmark. The evaluation was performed against reference calculations which solved the full EEM matrix, with a few exceptions. We give here a brief overview (Fig. 4), whereas the full details can be found in the Benchmark section of the Additional file 1. Both EEM Cutoff and EEM Cover are sufficiently accurate, but EEM Cutoff is slightly more accurate. Using a cutoff radius of 8 Å may lead to deviations of up to 0.015*e*, but on average less than 0.008*e*. Using a cutoff radius of 12 Å may lead to deviations of up to 0.008*e*, but on average less than 0.004*e*. The approaches are time efficient compared to Full EEM only when the molecule contains at least 10,000 atoms, but they are always more memory efficient.

Below we provide a few brief examples of uses for AtomicChargeCalculator in the form of case studies. These case studies are focused on a direct interpretation of the ACC results, and show how important hints about the reactivity of a molecule can be obtained in just a few seconds.

### Case study I: atomic charges and chemical reactivity in small drug-like molecules

*N*-acetyl-p-aminophenol, commonly known as paracetamol, is a widely used analgesic and antipyretic. Its mechanism of action is believed to be the inhibition of the protein cyclooxygenase 2, regulating the production of pro-inflammatory compounds [71]. The metabolic breakdown of paracetamol has been the subject of intense study, since it holds the key to both its therapeutic action and toxicity.

We calculated atomic charges in paracetamol using ACC. The geometry of the paracetamol molecule corresponded to the ideal coordinates [wwPDB CCD: TYL]. The default ACC settings were used. The computation took less than 1s, and the complete results are available on the ACC web page at http://ncbr.muni.cz/ACC/CaseStudy/Paracetamol.

**Fig. 5:**
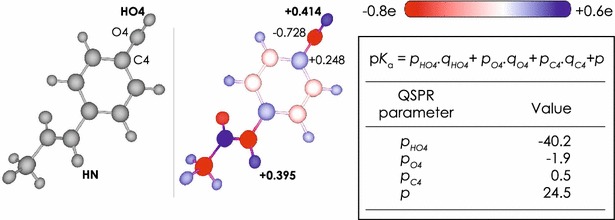
Atomic charges reveal reactive sites involved in the metabolic degradation of paracetamol. In the picture on the right, the atoms are colored according to their charge. The majority of metabolic degradation of paracetamol (glucuronidation, sulphonation, oxygenation) involves the phenolic (HO4) and amidic (HN) positions, the two most acidic protons in the paracetamol molecule (labels marked in bold) [72]. The relatively higher positive charge on these H atoms marks more active bonds compared to the rest of the molecule. In the equation of the QSPR model, q$_{HO4}$ is the charge on the phenolic H, q$_{O4}$ is the charge on the phenolic O, q$_{C4}$ is the charge on the C binding the phenolic OH, and p$_{HO4}$, p$_{O4}$, p$_{C4}$ and p are the parameters of the QSPR model, taken from the QSPR model 3d EEM Ouy2009_elem [58]

A quick analysis reveals that the phenolic H (position HO4 in Fig. 5) is the most acidic proton (highest positive charge) in the molecule, suggesting a faster and easier dissociation of this O–H bond. Indeed up to 90 % of metabolic degradation happens at position HO4 (glucuronidation, sulphonation) [72]. Additionally, up to 15 % of metabolic degradation involves oxidation at the phenolic (HO4) and amidic positions (HN) [72], the two most positive H in the paracetamol molecule. While paracetamol is a very small molecule with few polar sites, the same principle can be applied in reasoning out highly reactive sites in more complex molecules.

Having found out that the most probable dissociation site on the paracetamol molecule is the phenolic H, we were able then to calculate the acid dissociation constant p${K}_a$, a property which significantly affects the ability of the drug to cross cellular membranes and thus exert its therapeutic effect. For this purpose we used Quantitative Structure-Property Relationship (QSPR) modeling, as atomic partial charges have been shown to be successful QSPR descriptors in p${K}_a$ prediction [58, 73, 74], and QSPR models are available in literature for this purpose. Because the dissociating group on paracetamol is phenolic, we chose a QSPR model specifically developed for the prediction of p${K}_a$ in phenols [58], and which employed descriptors based on the EEM charges we computed in our interpretation of local reactivity in paracetamol. The necessary descriptors consisted of the partial charges on the phenolic oxygen (q$_{O4}$) and hydrogen (q$_{HO4}$), and on the carbon atom binding the phenolic group (q$_{C4}$). The equation and parameters of the QSPR model are given in Fig. 5.

We thus computed a p${K}_a$ value for paracetamol of 9.36, which is close to the experimental value of 9.38 [75]. The computed p${K}_a$ suggests that paracetamol is completely unionized at stomach pH, and only 1.1 % ionized at physiological pH, therefore highly efficient at crossing cellular membranes both via oral and intravenous delivery.

**Fig. 6:**
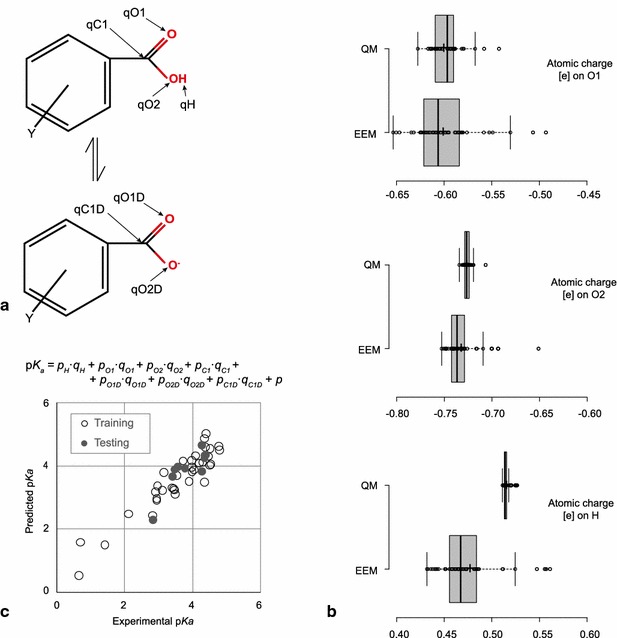
Limitations of EEM charges illustrated on a series of substituted benzoic acid derivatives. **a** Denotation of relevant atomic charges in the neutral and dissociated molecules. **b** The reference QM charges have a narrow spread despite the different position and wide range of electron donating or withdrawing effects of the subsituents. EEM charges are not accurate enough to reflect the small changes induced by different substituents. **c** The QSPR descriptors are EEM charges of the atoms of the carboxylic group in both the neutral ($q_H$, $q_{O1}$, $q_{O2}$, $q_{C1}$) and dissociated forms ($q_{O1D}$, $q_{O2D}$, $q_{C1D}$). The symbols $p_H$, $p_{O1}$, $p_{O2}$, $p_{C1}$, $p_{O1D}$, $p_{O2D}$, $p_{C1D}$, and *p* are parameters of the QSPR model. The graph displays the correlation between experimental $pK_a$ values, and the values predicted by one of the QSPR models developed in this study. EEM charge descriptors are sufficiently accurate for the prediction of dissociation constants of benzoic acid derivatives

While the above described approach was able to provide useful information for paracetamol, it is important to keep in mind that there are limitations to the accuracy of EEM charges. We illustrate such limitations on a series of benzoic acid derivatives. For this purpose, we downloaded the structures of 45 molecules representing benzoic acid derivatives from the NCI Open Database [76]. The data set contained benzoic acids with a wide range of donating and accepting substituents on the phenyl ring in o-, m- and p- positions (Fig. 6a; Additional file 1: Table S3). We chose only compounds for which $pK_a$ values were available in Physprop [77]. Furthermore, we did not include compounds with halogens because the EEM parameter set used in this particular case study cannot treat halogens.

We first wanted to know if and how the effect of different substituents in different positions on the phenyl ring is visible on the charge of the atoms of the carboxyl group. For this purpose, we used Gaussian [36] to compute reference QM atomic charges from a natural population analysis on the electron density obtained at the B3LYP/6-31G* level of theory. Despite the different nature and position of the substituents, the spread of reference QM charges for the atoms in the carboxyl group is very narrow (Fig.  6b). Specifically, the QM values for O1 are within 0.06*e*, the values for O2 within 0.01*e*, and the values for H within 0.01*e*. On the other hand, the EEM parameter set employed here is expected to reproduce the reference QM values within 0.09*e* [34]. Based on the documented accuracy, we do not expect EEM charges to reflect suitable changes based on the nature or position of the substituents. We used ACC to compute EEM charges for the benzoic acid derivatives, in the same manner as for paracetamol. The computation took less than 2s, and the complete results (structures, QM charges, EEM charges) are available on the ACC web page at http://ncbr.muni.cz/ACC/CaseStudy/BenzoicAcids.

Indeed, we found that EEM charges could not accurately reflect the QM spread for the charges on the carboxyl atoms (Fig. 6b). We then wondered if this accuracy, though unable to reflect suitable changes depending on the nature or position of the substituents, was sufficient to build acceptable QSPR models for $pK_a$ prediction. No suitable QSPR models are available for benzoic acids, but such models for aliphatic carboxylic acids have been reported [58, 78]. The descriptors used by these QSPR models consist of charges on the atoms of the carboxylic group in both the neutral ($q_H$, $q_{O1}$, $q_{O2}$, $q_{C1}$) and dissociated forms ($q_{O1D}$, $q_{O2D}$, $q_{C1D}$). We thus built QSPR models for benzoic acids based on these descriptors (Fig. 6c). We obtained the structures of the dissociated acids by removing the carboxylic H atoms. We computed EEM charges for the dissociated molecules using the same ACC setup, but setting the total charge for each molecule at $-$1. We then built QSPR models using multilinear regression. We performed a 5-fold cross-validation of the QSPR models, whereby, in each round, 35 randomly chosen molecules were used to train the model, and the remaining 10 molecules were used to validate the model. The QSPR model parameters and full details of the cross-validation procedure are given in the Additional file 1: Table S4. The models showed adequate predictive capability (Fig. 6c; Additional file 1: Table S4). On average, the mean absolute error during validation was 0.27 $pK_a$ units compared to experiment, suggesting that EEM charges can be used to predict dissociation constants for benzoic acids, despite their inability to reflect local changes caused by different substituents.

### Case study II: atomic charges and activity of antimicrobial peptides

**Fig. 7:**
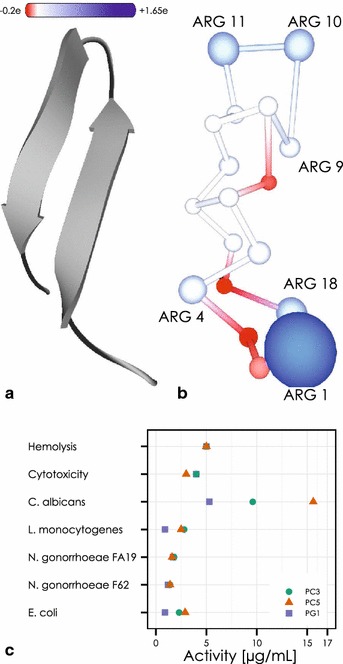
Residue charges in protegrin-1 (PG1) indicate relevant mutation sites. **a** Cartoon representation of the structure of PG1. **b** Schematic representation of the PG1 residues and their charge. Each residue position is represented by a *sphere*, the coordinates of which correspond to the average coordinates of all atoms in the residue. The *color* of the sphere is given by the residue charge, and the *size* of the sphere is proportional to the absolute charge. Cystein bridges are also displayed. The ARG residues at positions 4 and 9 are significantly less positive than the rest of the ARG residues, indicating that mutations at these positions may be less effective. **c** Antimicrobial activity (against five pathogens) and toxicity (cytotoxicity, hemolysis) of PG1 and two single mutants, namely PC3 (R4G) and PC5 (R10P) [83]. Although both mutants have increased activity, the mutation of ARG 4 in PC3 alters the antimicrobial activity against *C. albicans* significantly less than the mutation of ARG 10 in PC5, as predicted by the residue charges

Protegrins are a family of antimicrobial peptides active against a wide range of pathogens [79]. Protegrin-1 (PG1, Fig. 7) has been intensely studied for its potential in treating infections caused by antibiotic resistant bacteria [80–82]. PG1 shows activity against several pathogens, but also toxicity against the host. Useful mutations are those which maintain the antimicrobial activity, and at the same time reduce toxicity [83].

We calculated atomic charges in PG1 using ACC. The geometry of the PG1 molecule corresponded to a low energy NMR model [PDB: 1PG1] [84]. The system contained all H atoms expected at pH 6.5, as they were listed in the NMR model. The total molecular charge was +7, owing to the many ARG residues. The EEM parameter set used was EX-NPA_6-31Gd_gas [70], but it was necessary to add to this set EEM parameters for deuterium (D), because this element was present in the input file. The EEM parameters for D were identical to the parameters for H. The rest of the ACC default settings were kept. The computation took less than 1s, and the complete results are available on the ACC web page at http://ncbr.muni.cz/ACC/CaseStudy/Protegrin.

The calculation produced one set of atomic charges. PG1 contains 18 residues, and rather than analyzing atomic charges, we analyzed the residue charges, which are also reported by ACC (Fig.  7b). PG1 is special because of its high positive charge. It contains 6 ARG residues. However, not all have the same charge. In particular, ARG at positions 4 and 9 have the least positive charge (around +0.5*e*), whereas the rest have much higher positive charge (over +0.8*e*). Keeping in mind that these charges are likely affected by the polarizability exaggeration artifact of EEM described in the Computational details section, the results suggest that mutations of ARG into a neutral residue at positions 4 or 9 would have a lower effect than mutations at positions 1, 10, 11 or 18.

A literature search reveals that many mutants and derivatives of PG1 have been studied [83, 85–87]. In particular, Ostberg and Kastnessis [83] have logged the antimicrobial and toxic activity of sixty-two PG1 mutants and PG1-analogue peptides. Out of these, there are two single point mutants where one ARG was mutated into a neutral residue. These mutants are PC3 (R4G) and PC5 (R10P). The study found that, indeed, the mutation of ARG 4 in PC3 alters the antimicrobial activity against *C. albicans* significantly less than the mutation of ARG 10 of PC5 (Fig. 7c). Such biologically relevant insight can be gained by analyzing the residue charges on a single structure of PG1.

### Case study III: atomic charges and allostery of large biomacromolecular complexes

**Fig. 8:**
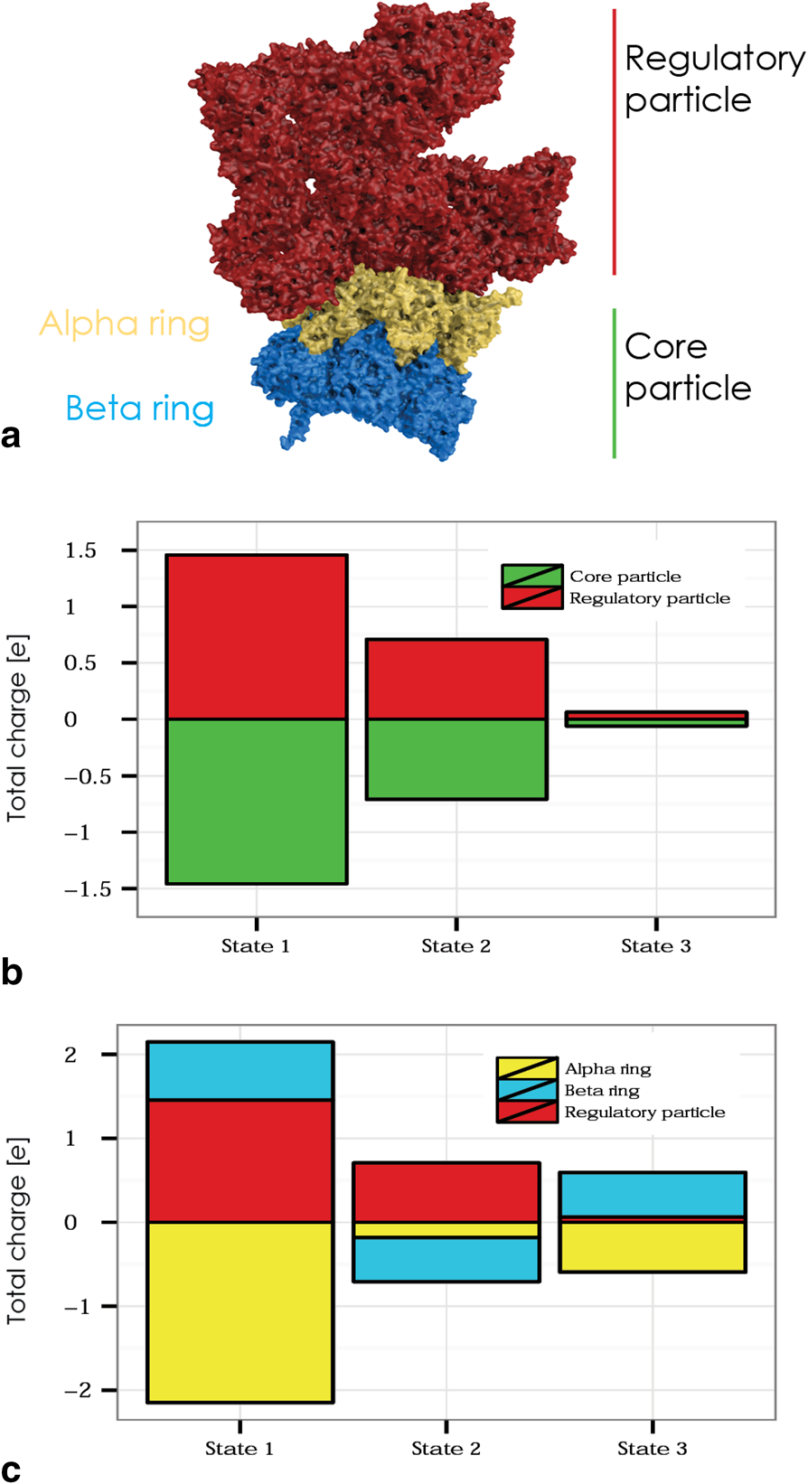
Using information about total subunit charge to track allosteric regulation in the 26S proteasome. **a** Surface representation of the structure of half of the 26S proteasome in state 1 [PDB: 4CR2]. The regulatory particle (*red*) enables the substrate to unfold and enter the core particle, which is made up of alpha (*yellow*) and beta rings (*blue*). **b** As the proteasome evolves from state 1 [PDB: 4CR2], through state 2 [PDB: 4CR3], to state 3 [PDB: 4CR4], the movement of the regulatory particle is translated into information which is exchanged with the core particle. The difference in total particle charge is a clear indication of regulation, despite the negligible movement observed in the core particle. **c** Information is transferred not just within each particle, but also vertically, between the alpha and the beta ring, and between the alpha ring and the regulatory particle. This suggests that vertical structures such as pairs of alpha-beta subunits may act in a correlated manner

The 26S proteasome is a large biomacromolecular complex which facilitates the targeted degradation of intracellular proteins, and thus plays an essential role in keeping protein homeostasis [88]. It consists of a core particle, made up of alpha rings and beta rings, controlled by regulatory particles, which are made up of a number of proteins (Fig. 8a). The proteasome is an intricate molecular machine which requires complex regulation to unfold and deubiquitylate the substrate, and push it through the catalytic machinery located in the beta rings [89]. Necessarily, the proteasome undergoes large conformational changes during its operation. However, due to its size, such changes are very difficult to study. Recent work in the field of cryo-electron microscopy [90] has led to the discovery of intermediate conformers during the initial binding of ubiquitylated substrates. While the conformational changes in the regulatory particle are easily distinguishable (average backbone atom RMSD 10.4 Å), the changes in the core particle are very subtle (average backbone atom RMSD 1.5 Å), due to the fact that all studied conformers refer to the initial phase of substrate binding.

Using ACC, we calculated atomic charges in these intermediate conformers of the 26S proteasome [PDB: 4CR2, 4CR3, 4CR4]. The default ACC settings were used. The computation took 130s, and the complete results are available on the ACC web page at http://ncbr.muni.cz/ACC/CaseStudy/Proteasome.

The calculation produced one set of atomic charges for each conformer of the 26S proteasome. Since the proteasome is very large, we analyzed the residue charges, which are also reported by ACC, and subsequently the charges for the various subunits that make up the proteasome (details regarding the charge analysis on subunits can be found in the section Case study III of the Additional file 1). The first observation was that, during the conformational changes from state 1, to state 2, and then to state 3, a significant amount of electron density is transferred between the core particle and the regulatory particle (Fig. 8b). This suggests that, even though there is no significant movement observed for the core particle, allosteric information is exchanged with the core particle, and this information can be tracked at the electrostatic level. The next observation is that significant information is disseminated not only horizontally (within the alpha ring, or within the beta ring), but also vertically. In the overall transition it appears that the alpha ring loses electron density to the regulatory particle. By checking the intermediate state 2 it is possible to see that there is also transfer between the alpha ring and beta ring (Fig. 8c). This vertical shuttling of electron density within the core particle suggests that the activity of alpha and beta subunits may cross-correlate. Such phenomena have indeed been reported. For example, alpha5 and beta1 may translocate together [91], while knockdown of alpha1 leads to loss of chymotrypsin activity associated with beta5 [92]. Further analysis can even yield the residues involved in the allosteric regulation, as those residues which exhibit a high variation in total charge (e.g., approximately 10 sites on the Rpn-13 regulatory subunit).

It is important to note that the structures used in the EEM calculation were incomplete. Specifically, due to the size of these molecular machines, the resolution of the structures was too low to distinguish H atoms or even parts of residues. No modifications were made to the structures of the proteasome conformers prior to the EEM calculation. Thus, the charge distribution of each conformer is not expected to be physically relevant taken on its own. Moreover, the results are very likely affected by the polarizability exaggeration artifact of EEM, discussed in the Computational details section. Therefore, the analysis here focused on how the amount of charge in functional parts of the proteasome changes with the conformation. This case study shows how a brief calculation using only a crude structural approximation can give insight regarding allosteric regulation in large biomolecular complexes.

## Conclusions

We present AtomicChargeCalculator (ACC), a web-based application for the calculation and analysis of atomic charges which respond to changes in molecular conformation and chemical environment. ACC also provides interactive facilities for statistical analysis and comparison of the results. We illustrate how direct analysis of atomic charges can give basic information about chemical reactivity in paracetamol, and how residue charges hold clues about biochemical relevance in the antimicrobial peptide protegrin-1. Additionally, ACC provides molecular structure files containing atomic charges, which can be used in further modelling studies. We illustrate how such data can be used for p${K}_a$ calculation using QSPR models. Another advantage of ACC is that it can handle any type of molecular system, regardless of size and chemical complexity, from drug-like molecules to biomacromolecular complexes with hundreds of thousands of atoms. We show how the direction and intensity of allosteric regulation can be tracked in large biomacromolecular systems like the proteasome even in the absence of high resolution structures. ACC thus caters to all fields of life sciences, from drug design to nano-carriers. AtomicChargeCalculator is freely available online at http://ncbr.muni.cz/ACC.

## Availability and requirements

- *Project name* AtomicChargeCalculator
- *Project home page*http://ncbr.muni.cz/ACC
- *Operating system(s)* Web server - platform independent. Command line application—Windows, Linux, Mac OS
- *Programming language* C#
- *Other requirements* For the web-server - modern internet browser with JavaScript enabled, WebGL support for 3D visualization. For the command line application - NET 4.0 for Windows based systems, Mono framework 3.10 or newer (http://www.mono-project.com) for other OS.
- *License* ACC license for the downloadable command line version.
- *Any restrictions to use by non-academics* Free of charge. No login requirement for running or accessing the results in the web server.

## Additional file

10.1186/s13321-015-0099-x Full computational details, benchmark and limitations of ACC, along with supporting information for case studies I and III.
