# Supplementary material for: AtomicChargeCalculator: interactive web-based calculation of atomic charges in large biomolecular complexes and drug-like molecules
Source: J Cheminform. 2015 Oct 22;7:50. doi: 10.1186/s13321-015-0099-x (PMC4613891; doi:10.1186/s13321-015-0099-x)
Supplement: Supplementary file 1 — 10.1186/s13321-015-0099-x Full computational details, benchmark and limitations of ACC, along with supporting information for case studies I and III. [file 13321_2015_99_MOESM1_ESM.pdf]

Additional file

AtomicChargeCalculator: interactive web-based calculation of atomic charges in large biomolecular complexes and drug like molecules

Crina-Maria Ionescu[1], David Sehnal[1,2,3], Francesco L Falginella[1], Purbaj Pant[2], Lukáš Pravda[1,2], Tomáš Bouchal[1,2], Radka Svobodová Vařeková[1,2], Stanislav Geidl[1,2], Jaroslav Koča[1,2,*]

[1]CEITEC - Central European Institute of Technology, Masaryk University Brno, Kamenice 5, 625 00 Brno, Czech Republic
[2]National Centre for Biomolecular Research, Faculty of Science, Masaryk University, Kotlářská 2, 611 37 Brno, Czech Republic
[3]Faculty of Informatics, Masaryk University Brno, Botanická 68a, 602 00 Brno, Czech Republic

* Corresponding author:
JK: Tel: +420 54949 4947; Fax: +420 54949 2556; Email: Jaroslav.Koca@ceitec.muni.cz

The authors wish it to be known that, in their opinion, the first 2 authors should be regarded as joint First Authors.

Web address: `http://ncbr.muni.cz/ACC`

Computational details

EEM

Given the 3D structure of a molecule with N atoms and total charge Q, EEM estimates the partial atomic charges $q_1 \ldots q_N$ and the average molecular electronegativity χ via a set of coupled linear equations:

$$\begin{pmatrix} B_1 & \frac{k}{r_{1,2}} & \cdots & \frac{k}{r_{1,N}} & -1 \\ \frac{k}{r_{2,3}} & B_2 & \cdots & \frac{k}{r_{2,N}} & -1 \\ \vdots & \vdots & \ddots & \vdots & \vdots \\ \frac{k}{r_{N,1}} & \frac{k}{r_{N,2}} & \cdots & B_N & -1 \\ 1 & 1 & \cdots & 1 & 0 \end{pmatrix} \cdot \begin{pmatrix} q_1 \\ q_2 \\ \vdots \\ q_N \\ \chi \end{pmatrix} = \begin{pmatrix} -A_1 \\ -A_2 \\ \vdots \\ -A_N \\ Q \end{pmatrix} \tag{1}$$

where $r_{i,j}$ is the distance between atoms i and j, and A_i and B_i are EEM parameters for atom i. The factor k, although originally a unit conversion factor [1], has been exploited in some EEM models as an adjustable parameter (e.g. [2, 3]). ACC calculates the interatomic distances $r_{i,j}$ based on the atomic positions in the molecular structure file. The user is required to provide the total charge, or ACC assumes the molecule is neutral (total molecular charge is 0). EEM parameters for each atom type (e.g., carbon, oxygen) present in the molecule are read from a set of EEM parameters suitable for the molecule in question. Many parameter sets published in literature are available in ACC. Alternatively, the user may provide a custom set using ACC's dedicated XML template.

EEM Cutoff

For each atom in the molecule, ACC generates a fragment made up of all atoms within a cutoff radius R of the original atom. The values of the inter-atomic distances and EEM parameters are obtained in the same way as when solving the full EEM matrix. The total fragment charge Q_F is a quota of the total molecular charge Q, proportional to the number of atoms in the fragment (N_F), and irrespective of the nature of these atoms:

$$Q_F = \frac{Q \cdot N_F}{N} \tag{2}$$

Then ACC solves the EEM matrix equation for this fragment, and returns the charge for the atom used when generating the fragment. The same procedure is applied for all fragments, obtaining a set of charges for all the atoms in the molecule. Then, each atomic charge q_i is corrected by the addition of:

$$\frac{Q - \sum_{i=1}^{N} q_i}{N} \tag{3}$$

so that the sum of all atomic charges equals the total molecular charge Q.

In total, for a molecule with N atoms, the EEM Cutoff approach solves N smaller EEM matrices, corresponding to a set of N overlapping fragments of the original molecule. Compared to solving the full EEM matrix equation in the original EEM approach, EEM Cutoff reduces the time complexity of the calculation to $\mathcal{O}((R^3)^3N + R^3N\ log\ N)$, where N is the number of atoms and R is the cutoff radius parameter. The R^3 factor comes from the fact that a spherical fragment of radius R can contain at most R^3 atoms. Therefore, solving the EEM matrix requires $\mathcal{O}(R^9)$ steps. The $\mathcal{O}(R^3N\ log\ N)$ term represents the complexity of finding the atoms that belong to each fragment. However, for most molecules, there are on average $\mathcal{O}(R^2)$ atoms in each fragment, effectively reducing the complexity to $\mathcal{O}((R^2)^3N + R^2N\ log\ N)$. The space complexity of the approach is $\mathcal{O}((R^3)^2 + N\ log\ N)$, where R^3 again comes from the number of atoms

in the fragment (effectively R^2 in most practical cases), and represents the memory required to store the reduced EEM matrix. The $N\ log\ N$ term represents the memory requirements of the spatial lookup data structure. EEM Cutoff is efficient for molecules containing at least several thousands of atoms.

EEM Cover

The EEM Cover approach builds on the principles of EEM Cutoff to split the EEM matrix into smaller matrices. However, EEM Cover generates fragments only for a subset of atoms in the molecule. The procedure selects fragment-generating atoms so that: (i) no two such atoms are connected to each other, and (ii) each atom in the molecule has at least one neighbor (within two bonds) which was selected. This procedure ensures that each atom in the molecule will eventually contribute to at least one fragment, and thus the entire volume of the molecule is covered. ACC solves the EEM matrix equation for each fragment, and returns a list of charge contributions for all atoms encountered in the calculations. The charge on each atom in the molecule is then computed as the sum of its charge contributions from all fragments where the atom is present. Further, each atomic charge q_i is corrected by the addition of:

$$\frac{Q - \sum_{i=1}^{N} q_i}{N} \tag{4}$$

so that the sum of all atomic charges equals the total molecular charge Q.

Given the same cutoff radius R, EEM Cover has the same asymptotic complexity as EEM Cutoff. However, thanks to the procedure of selecting fragment-generating atoms (which requires $\mathcal{O}(N\ log\ N)$ steps), the number of EEM matrices that need to be solved during EEM Cover is reduced by at least 50% compared to EEM Cutoff (since, for each atom, at least one neighbor will not be selected).

Default settings

Total charge

ACC assumes that all uploaded molecules are neutral unless specified otherwise.

EEM parameter set

ACC choses the default EEM parameter set based on the number of atoms in the largest uploaded molecule, and the chemical elements of atoms in all the uploaded molecules. Specifically, if the largest uploaded molecule contains 255 atoms or fewer, a set with the target organic molecules is chosen. If the largest uploaded molecule contains more than 255 atoms, a set with the target biomolecules is chosen. The chosen set must cover all the chemical elements in all the uploaded molecules, and if more such sets are available, the one with the lowest priority is chosen. If no set covers all chemical elements in the uploaded molecules, then no default set is chosen.

Computation method

ACC chooses the default computation method based on the number of atoms in the largest uploaded molecule. Specifically, if the largest uploaded molecule contains 30,000 atoms or fewer, the default method will be Full EEM, in double precision (64-bit numbers) and with inclusion of potential water molecules. If the largest uploaded molecule contains more than 30,000 atoms, the default method will be EEM Cover with a cutoff radius of 12Å, in double precision and with inclusion of potential water molecules.

Benchmark

The reliability of the EEM Cutoff and EEM Cover approaches was evaluated in a benchmark against the classical procedure where the entire matrix equation is solved (Full EEM). The purpose of the benchmark was twofold: to evaluate the accuracy of the results produced by the newly developed approaches, and to quantify the reduction in computational resources required for these calculations. The structure of each molecule used in the benchmark was downloaded from the PDB, and no additional modifications were performed (adding H, optimizing geometry, etc.). For all jobs, the total molecular charge was 0, and the EEM parameter set employed was EX-NPA_6-31Gdd_gas [3].

Table S1: Accuracy of EEM Cutoff and EEM Cover, expressed as RMSD $[e]$ to reference calculations.

PDB ID	Total atoms	EEM Cutoff 8Å		EEM Cutoff 12Å		EEM Cover 8Å		EEM Cover 12Å	
		Single	Double	Single	Double	Single	Double	Single	Double
1lfg	5,884	0.014	0.014	0.008	0.008	0.015	0.015	0.008	0.008
3dtu	13,961	0.007	0.007	0.004	0.004	0.007	0.007	0.004	0.004
1tye	21,016	0.006	0.006	0.003	0.003	0.006	0.006	0.003	0.003
1occ	28,712	0.004	0.004	0.002	0.002	0.004	0.004	0.002	0.002
3unb*	99,236	0.005	0.005	0.002	0.002	0.006	0.006	0.002	0.002
4o9y*	197,663	0.005	0.005	0.002	0.002	0.005	0.005	0.002	0.002
4u1u*	288,328	0.008	0.008	0.003	0.003	0.007	0.007	0.003	0.003
4u6f*	411,881	0.007	0.007	0.003	0.003	0.007	0.007	0.003	0.003
4v8p*	511,395	0.008	0.008	0.003	0.003	0.008	0.008	0.003	0.003
4v99*	588,120	0.005	0.005	0.002	0.002	0.005	0.005	0.002	0.002

The terms Single and Double refer to the representation of the matrix using 32-bit or 64-bit precision numbers, respectively. The RMSD is calculated by comparison against the reference calculation Full EEM, with Double precision. *Due to the limitation on computational resources, for some molecules the reference calculation was EEM Cutoff, with a cutoff radius of 17Å.

The results in Table S1 show that the accuracy of both EEM Cutoff and EEM Cover increases with the cutoff radius, which is to be expected. For the typical application of EEM Cutoff and EEM Cover, the maximum expected deviation per atom is within $0.01e$ of the corresponding Full EEM calculation. Another observation is that for EEM Cutoff and EEM Cover, the precision used to represent the EEM matrix does not affect the accuracy.

The results in Table S2 suggest that there is no advantage in using the EEM Cover approximation for molecules with fewer than 10,000 atoms. However, with increasing molecular size, the reduction in run time and memory usage becomes significant. The EEM Cover approximation can decrease the requirements for computational resources by one order of magnitude, thus allowing calculations for much larger molecules to run on conventional desktop machines. For example, in the case of the molecules with PDB IDs 3unb and 4v99, the Full EEM would require 73GB and 2.5TB memory, respectively, just to represent the EEM matrix using 64-bit precision numbers.

Table S2: Comparison of run time and peak memory usage between Full EEM and EEM Cover with cutoff radius 8Å and 12Å.

PDB ID	Total atoms	Full EEM		EEM Cover 8Å		EEM Cover 12Å	
		time [s]	memory [GB]	time [s]	memory [GB]	time [s]	memory [GB]
1lfg	5,884	3	0.50	2	0.20	4	0.20
3dtu	13,961	24	1.80	4	0.30	9	0.30
1tye	21,016	72	3.80	5	0.40	10	0.50
1occ	28,712	175	6.80	6	0.50	15	0.50
3unb*	99,236	-	-	20	1.20	50	1.40
4o9y*	197,663	-	-	38	2.23	85	2.53
4u1u*	288,328	-	-	56	2.55	124	2.96
4u6f*	411,881	-	-	80	3.37	188	3.70
4v8p*	511,395	-	-	99	4.04	233	4.60
4v99*	588,120	-	-	115	4.40	279	4.90

The calculations ran on a machine with the following configuration: Intel i7-3770 3.4Ghz processor, 32GB RAM, SSD drive, using Microsoft Windows Server 2008 R2 and .NET Framework 4.5.2. Each calculation was repeated 7 times. The highest and lowest durations were removed, and the results were averaged over the remaining 5 runs. In all calculations, the EEM matrix was represented using 64-bit precision numbers.

Limitations

The most important limitation of ACC is inherent to the concept of atomic partial charges. A single number can give an idea about whether there is more electron density around some atoms compared to others, but it cannot characterize the actual distribution of electron density in the space between the atomic nuclei. Thus, all properties which flow from this distribution (e.g., multipole moments) are generally not well described using atomic partial charges of any sort [4]. Further, atomic charge definitions have their own specific limitations (e.g., AIM, NPA and Mulliken charges give poor estimates of electrostatic potentials [5]).

The second limitation relates to the empirical nature of the EEM approach. EEM relies on empirical parameters fitted to reference QM data. As such, when employing a particular set of EEM parameters, it is important to consider the nature of the reference QM data, as well as the particular fitting strategy used in the development of the set of EEM parameters. Moreover, EEM incorrectly predicts superlinear scaling of the polarizability with increasing molecule size, leading to underestimation of multipole moments for extended systems like biomacromolecules. In general, one cannot expect that EEM charges will outperform QM charges. Similarly, EEM charges cannot be expected to outperform other empirical charges specifically developed for a certain purpose, as is the case of atomic partial charges optimized for use in simulations with specific force fields (e.g., RESP charges used with Amber force fields, Gromos-like charges used with Gromos force fields, etc.). While extensions to EEM have been proposed [6–14], ACC currently implements the classical EEM formalism, due to wider accessibility of EEM parameters for this formalism. One specific limitation exists for the EEM Cover approximation in the event that no EEM parameters are available for some atom types present in the molecule, and the connectivity network in that area of the molecular structure is sparse (typically, when H are missing, and some atoms appear to be bound only to an atom without EEM parameters). In this case, no charges will be calculated for these sparsely connected atoms, even if EEM parameters are available.

The third limitation relates to each particular molecular structure under investigation. The

responsibility for suitable input for ACC, i.e., ensuring the structure is complete and in a relevant conformation, and the total molecular charge is reasonable, currently lies with the user. Providing inappropriate input may produce charges which are not representative of the particular phenomenon under study. Further, the size of the input file is limited to 50MB.

Case Study I

Table S3: List of NSC numbers, molecule names, and pK_a values for the benzoic acid derivatives used in this case study.

NSC	Name	pK_a	Predicted pK_a i	ii	iii	iv	v
NSC 133453	2,4,6-trinitrobenzoic acid	0.65	0.55	0.55	0.52	0.60	0.57
NSC 13564	2,4-dihydroxybenzoic acid	3.11	3.23	3.20	3.21	3.22	3.14
NSC 147400	2,4,5-trimethylbenzoic acid	4.38	4.23	4.30	4.33	4.26	4.18
NSC 15797	4-formylbenzoic acid	3.77	3.92	3.95	3.91	3.92	3.78
NSC 16631	3,4-dihidroxybenzoic acid	4.26	3.82	3.85	3.82	3.83	3.68
NSC 16632	4-propoxybenzoic acid	4.78	4.58	4.60	4.61	4.56	4.58
NSC 16634	2-hydroxy-4-methylbenzoic acid	3.40	3.65	3.62	3.66	3.61	3.57
NSC 17831	2-acetamidobenzoic acid	3.40	3.20	3.27	3.30	3.27	3.16
NSC 180	2-hydroxybenzoic acid	2.97	3.35	3.33	3.35	3.31	3.17
NSC 181	2-hydroxy-3,5-dinitrobenzoic acid	0.70	1.62	1.63	1.57	1.75	1.69
NSC 183	2-hydroxy-5-nitrobenzoic acid	2.12	2.50	2.50	2.48	2.55	2.46
NSC 20083	4-isopropylbenzoic acid	4.35	4.83	4.84	4.86	4.78	4.77
NSC 2129	4-hydroxy-3,5-dimethoxybenzoic acid	4.34	4.25	4.26	4.24	4.27	4.32
NSC 215211	2-(phenylamino)benzoic acid	3.99	3.73	3.76	3.81	3.75	3.87
NSC 2193	2-methylbenzoic acid	3.98	3.84	3.93	3.95	3.89	3.68
NSC 2214	3-methylbenzoic acid	4.27	4.63	4.65	4.65	4.58	4.48
NSC 2215	4-methylbenzoic acid	4.37	4.58	4.59	4.59	4.53	4.41
NSC 22948	3,5-dihidroxybenzoic acid	4.04	3.87	3.90	3.87	3.86	3.71
NSC 243713	2,4-dinitrobenzoic acid	1.42	1.54	1.54	1.49	1.63	1.64
NSC 246039	4-phenoxybenzoic acid	4.52	4.54	4.55	4.55	4.54	4.62
NSC 27224	2,5-dihidroxybenzoic acid	2.95	2.92	2.92	2.90	3.26	2.98
NSC 27435	2,3-dihydroxybenzoic acid	2.91	3.17	3.15	3.16	3.16	3.06
NSC 3778	2-methoxybenzoic acid	3.90	3.50	3.45	3.50	3.44	3.43
NSC 38518	2-hydroxy-5-methylbenzoic acid	3.15	3.79	3.75	3.79	3.74	3.72
NSC 39656	2-phenoxybenzoic acid	3.53	3.68	3.64	3.70	3.65	3.81
NSC 3987	4-hydroxy-3-methoxybenzoic acid	4.51	3.99	4.03	4.01	4.00	3.91
NSC 406186	2-acetoxybenzoic acid	3.49	3.06	3.06	3.10	3.06	3.05
NSC 407533	2,3-dimethylbenzoic acid	3.72	4.05	4.13	4.15	4.09	3.95
NSC 43744	3,5-dimethoxybenzoic acid	3.97	4.15	4.19	4.17	4.14	4.09
NSC 4802	4-tert-butylbenzoic acid	4.40	4.99	5.00	5.02	4.94	4.99
NSC 4961	4-hydroxybenzoic acid	4.54	4.05	4.07	4.05	4.03	3.84
NSC 50796	2-hydroxy-3-methylbenzoic acid	2.95	2.95	2.94	2.96	2.95	2.94
NSC 55746	3-hydroxybenzoic acid	4.30	4.11	4.13	4.10	4.08	3.91
NSC 60713	3,4-dinitrobenzoic acid	2.82	2.48	2.52	2.43	2.60	2.52
NSC 6306	4-cyanobenzoic acid	3.55	3.97	3.99	3.96	3.97	3.85
NSC 6316	2,4-dimethoxybenzoic acid	4.36	3.48	3.43	3.48	3.45	3.52
NSC 76051	2-phenylbenzoic acid	3.46	3.74	3.85	3.88	3.77	3.75
NSC 7707	4-nitrobenzoic acid	3.44	3.29	3.33	3.27	3.35	3.20
NSC 7721	3,4-dimethoxybenzoic acid	4.36	4.31	4.34	4.32	4.32	4.32
NSC 7926	4-methoxybenzoic acid	4.47	4.28	4.30	4.29	4.26	4.16
NSC 8705	4-ethoxybenzoic acid	4.80	4.48	4.50	4.50	4.46	4.43
NSC 8732	3,5-dinitrobenzoic acid	2.82	2.32	2.38	2.28	2.44	2.32
NSC 9264	3-methoxybenzoic acid	4.09	4.32	4.33	4.32	4.29	4.22
NSC 94437	2-[(2,3-dimethylphenyl)amino]benzoic acid	4.20	3.99	4.02	4.08	4.00	4.19
NSC 9801	3-nitrobenzoic acid	3.46	3.27	3.29	3.23	3.31	3.20

The experimental pK_a values were obtained from Physprop. The labels i-v refer to the rounds of cross validation. In each round, 10 molecules were randomly chosen to serve for validation (testing), while the remaining 35 molecules served for training the QSPR model.

Table S4: Parameters and quality of the QSPR models developed in this case study.

Round	QSPR parameters								MAE (pK_a units)	
	p_H	p_{O1}	p_{O2}	p_{C1}	p_{O1D}	p_{O2D}	p_{C1D}	p	training	testing
i	-16.351	152.022	19.328	-109.696	-141.202	-5.875	91.188	37.058	0.299	0.261
ii	-17.280	137.650	24.436	-101.777	-127.740	-4.870	82.480	41.902	0.282	0.286
iii	-20.154	136.799	25.277	-100.112	-126.695	-4.730	81.972	43.470	0.291	0.277
iv	-8.357	140.648	10.209	-106.491	-133.069	-6.470	88.391	24.186	0.296	0.256
v	-4.824	169.273	5.505	-135.629	-158.839	-13.386	116.965	16.728	0.291	0.279

Each model is trained on a set of 35 molecules, and then validated on a set of 10 molecules which were not used for training. The quality of the QSPR model obtained in each round of the cross validation procedure is evaluated by the mean absolute error (MAE) between the experimental and predicted pK_a. The QSPR descriptors consisted of atomic charges, which are available online at `http://ncbr.muni.cz/ACC/CaseStudy/BenzoicAcids`.

Case Study III

The 26S proteasome structure used in this study represents half of the biological assembly, and is made up of 33 subunits. Out of these, 14 subunits form the core particle (subunits beta1-7 and alpha1-7), while 19 subunits form the regulatory particle. Table S5 summarizes these subunits and their charge related properties in states 1 to 3.

Table S5: Subunits of the 26S proteasome and their total charge in states 1 to 3.

Subunit	Total charge [e]		
	State 1	State 2	State 3
beta1	-1.176	-0.453	-1.107
beta2	1.076	0.463	0.988
beta3	-0.547	-0.975	-0.123
beta4	1.283	0.941	0.415
beta5	1.178	0.639	1.495
beta6	0.057	-0.267	0.360
beta7	-1.180	-0.876	-1.496
alpha1	0.338	0.864	-0.168
alpha2	1.302	2.324	3.391
alpha3	-3.243	-2.229	-2.523
alpha4	0.315	1.252	0.058
alpha5	-1.620	-2.661	-2.416
alpha6	-0.871	-0.587	-0.372
alpha7	1.630	0.857	1.436
rpt1	2.756	2.299	2.118
rpt2	-1.295	-2.006	0.325
rpt6	-0.357	-0.507	-0.701
rpt3	-1.740	-0.495	-2.234
rpt4	1.712	2.575	2.545
rpt5	0.322	0.796	0.109
rpn2	-2.357	-2.154	-2.300
rpn9	3.683	2.338	2.042
rpn5	0.887	0.037	-1.184
rpn6	2.295	0.744	1.219
rpn7	0.055	-0.641	-0.506
rpn3	0.989	0.992	-1.039
rpn12	-1.282	-0.710	0.529
rpn8	-1.773	-1.352	1.046
rpn11	2.220	1.970	-0.216
rpn10	-1.550	-1.003	-0.232
rpn13	-1.088	-0.972	-0.654
sem1	-0.883	-1.086	-1.052
rpn1	-1.138	-0.117	0.247

For each subunit, the total charge was calculated as the algebraic sum of atomic charges for all atoms in that subunit. These are available online at `http://ncbr.muni.cz/ACC/CaseStudy/Proteasome`.

Bibliography

[1] Mortier W J, Ghosh S K, Shankar S: Electronegativity-equalization method for the calculation of atomic charges in molecules. Journal of the American Chemical Society 1986, 108(15): 4315-4320.

[2] Yang Z, Shen E: Molecular electronegativity in density functional theory (I) - Direct calculation of atomic charges in a molecule via electronegativity equalization principle SCIENCE CHINA Chemistry 1995, 38(5): 521-528

[3] Ionescu CM, Geidl S, Svobodová Vařeková R, Koča J: Rapid calculation of accurate atomic charges for proteins via the electronegativity equalization method. J Chem Inf Model 2013, 53:2548–2558.

[4] Kramer C, Spinn A, Liedl KR: Charge Anisotropy: Where Atomic Multipoles Matter Most. J Chem Theory Comput 2014.

[5] Sigfridsson E, Ryde U: Comparison of methods for deriving atomic charges from the electrostatic potential and moments. J Comput Chem 1998, 19:377–395.

[6] Baekelandt B, Mortier W, Lievens J: Probing the reactivity of different sites within a molecule or solid by direct computation of molecular sensitivities via an extension of the electronegativity equalization. J 1991, 113:6730–6734.

[7] Rappé AK, Goddard III WA: Charge Equilibration for Molecular Dynamics Simulations. J Phys Chem 1991, 95:3358–3363.

[8] York DM, Yang W: A chemical potential equalization method for molecular simulations. J Chem Phys 1996, 104:159.

[9] Yang Z-Z, Wang C-S: Atom–Bond Electronegativity Equalization Method. 1. Calculation of the Charge Distribution in Large Molecules. J Phys Chem A 1997, 101:6315–6321.

[10] Njo SL, Fan J, Van De Graaf B: Extending and simplifying the electronegativity equalization method. In Journal of Molecular Catalysis A: Chemical. Volume 134; 1998:79–88.

[11] Dias LG, Shimizu K, Farah JPS, Chaimovich H: A simple method for the fast calculation of charge redistribution of solutes in an implicit solvent model. Chem Phys 2002, 282:237–243.

[12] Chaves J, Barroso JM, Bultinck P, Carbó-Dorca R: Toward an alternative hardness kernel matrix structure in the Electronegativity Equalization Method (EEM). J Chem Inf Model 2006, 46:1657–1665.

[13] Ouyang Y, Ye F, Liang Y: A modified electronegativity equalization method for fast and accurate calculation of atomic charges in large biological molecules. Phys Chem Chem Phys 2009, 11:6082–6089.

[14] Verstraelen T, Van Speybroeck V, Waroquier M: The electronegativity equalization method and the split charge equilibration applied to organic systems: Parametrization, validation, and comparison. J Chem Phys 2009, 131.
